# Supplementary material for: Mechanism of allosteric inhibition of human p97/VCP ATPase and its disease mutant by triazole inhibitors
Source: Commun Chem. 2024 Aug 9;7:177. doi: 10.1038/s42004-024-01267-3 (PMC11316111; doi:10.1038/s42004-024-01267-3)
Supplement: Supplementary file 2 — Supplementary Information [file 42004_2024_1267_MOESM2_ESM.pdf]

SUPPLEMENTARY FIGURES

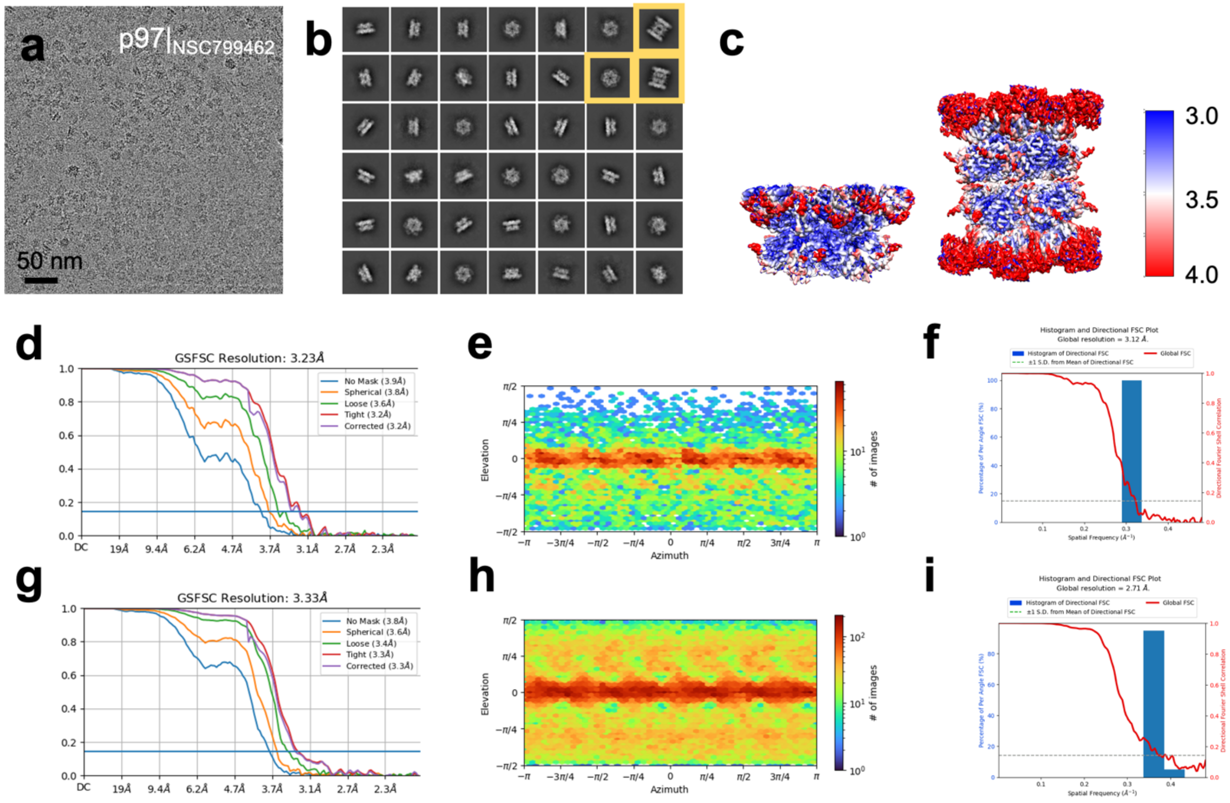

Supplementary Fig. 1 | Single-particle cryo-EM analysis of p97 with the lead compound NSC799462. **a,** Electron micrograph of the cryogenic p97|$_{\mathrm{NSC799462}}$. Scale bar indicates 50 nm. **b,** Representative two-dimensional (2D) class averages of single-particle images of p97|$_{\mathrm{NSC799462}}$. Class averages of 2D projections of the dodecamer are highlighted in orange. Box side length is 36.9 nm. **c,** Local resolution map of the three-dimensional (3D) reconstructions of p97|$_{\mathrm{NSC799462}}$ hexamer (left) and dodecamer (right), respectively. **d** and **g,** Fourier shell correlation (FSC) plots of the two 3D reconstructions. Upper and lower plots are for the hexameric and dodecameric p97|$_{\mathrm{NSC799462}}$, respectively. The resolutions were determined using gold standard FSC criteria at 0.143 cutoff. **e** and **h,** Euler angle distributions of the particle images for the reconstructions of the hexamer and dodecamer, respectively. **f** and **i**, 3DFSC plots of the reconstructions of the hexamer and dodecamer, respectively.

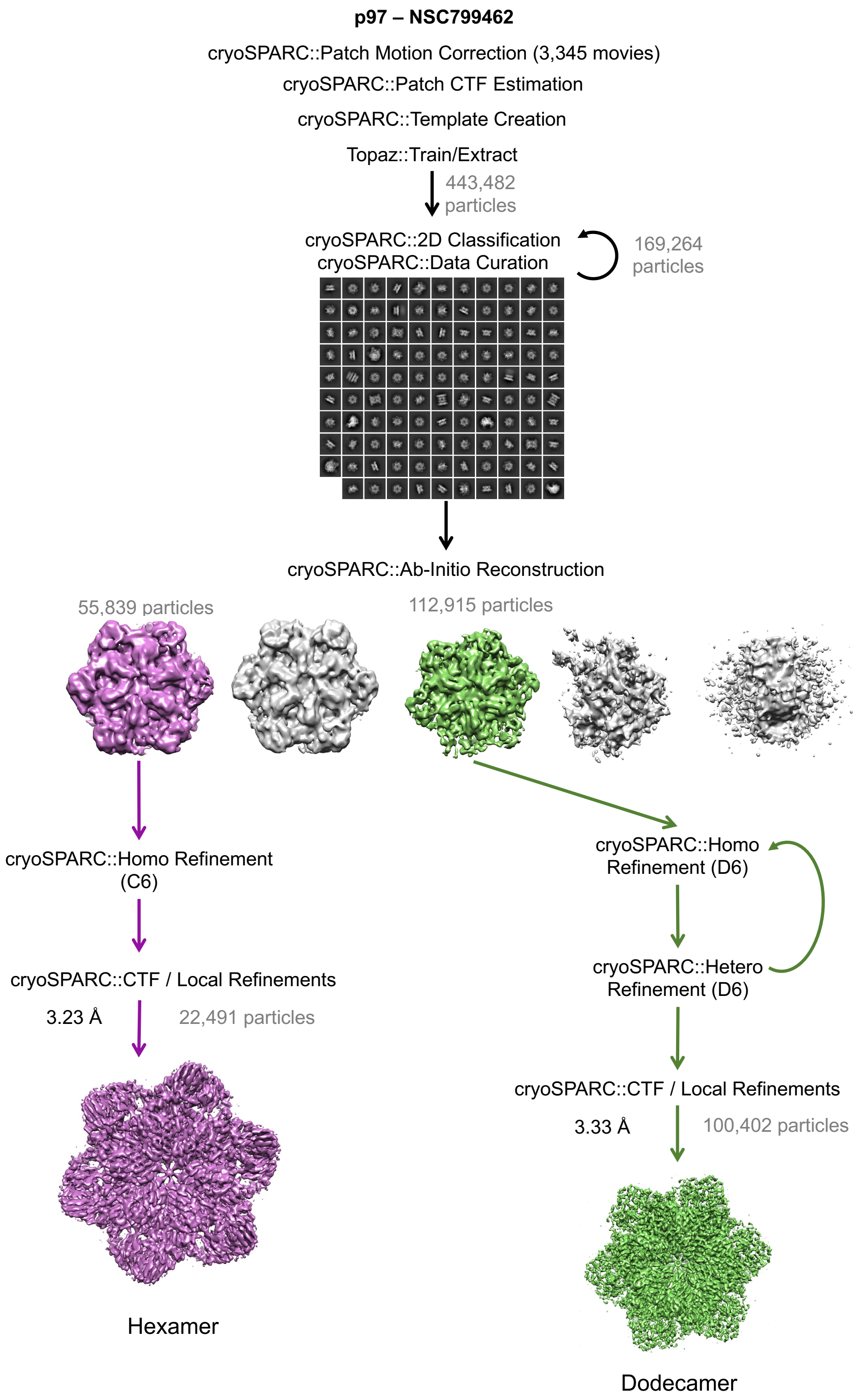

Supplementary Fig. 2 | Flowchart of single-particle cryo-EM image analysis of p97|$_{NSC799462}$.

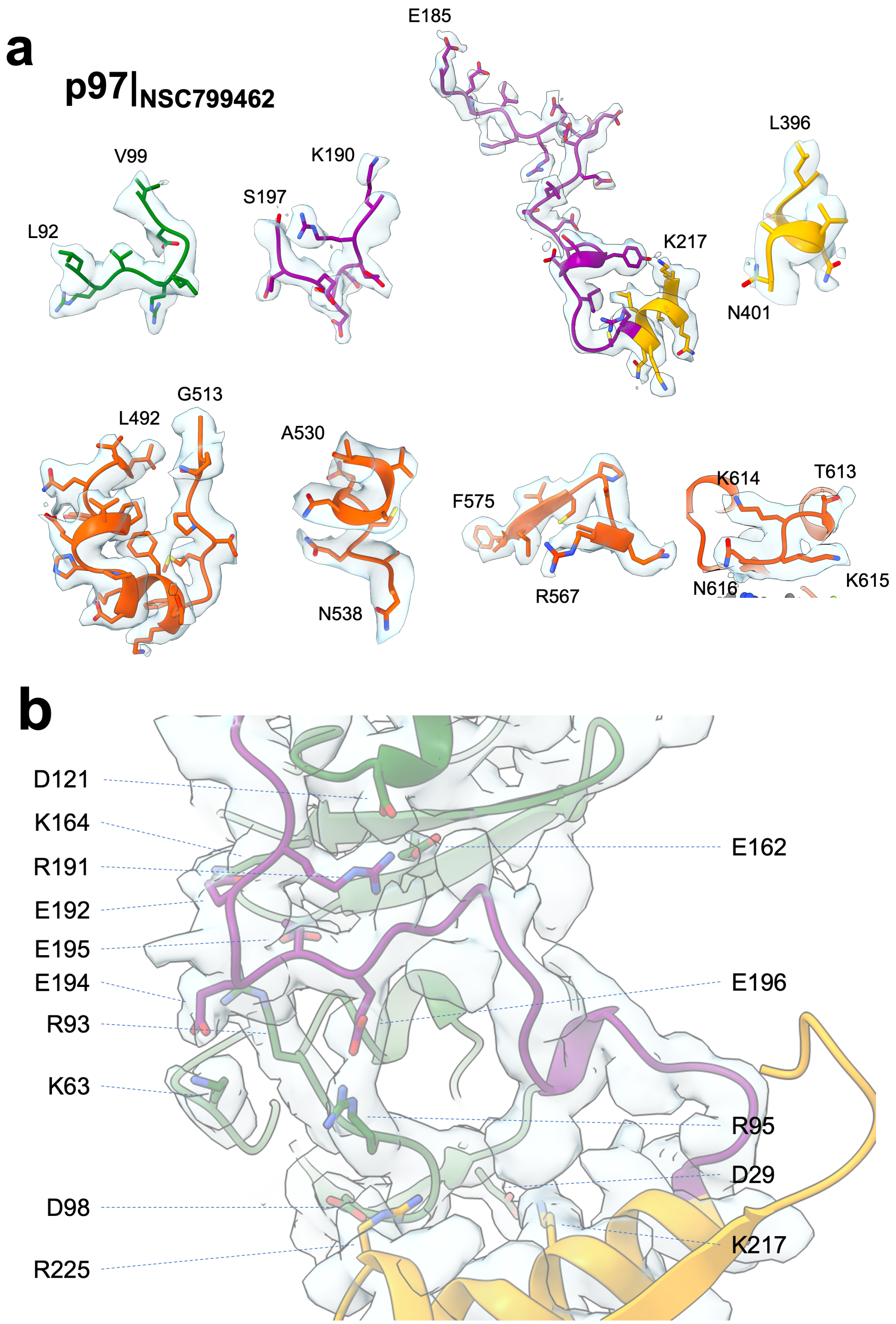

Supplementary Fig. 3 | Model fitting of the cryo-EM density map of p97|$_{NSC799462}$. **a,** Model-map fitting of the areas mentioned. Light blue surfaces represent cryo-EM densities. **b,** Fitting shown in the orientation corresponding to **Fig. 4b**.

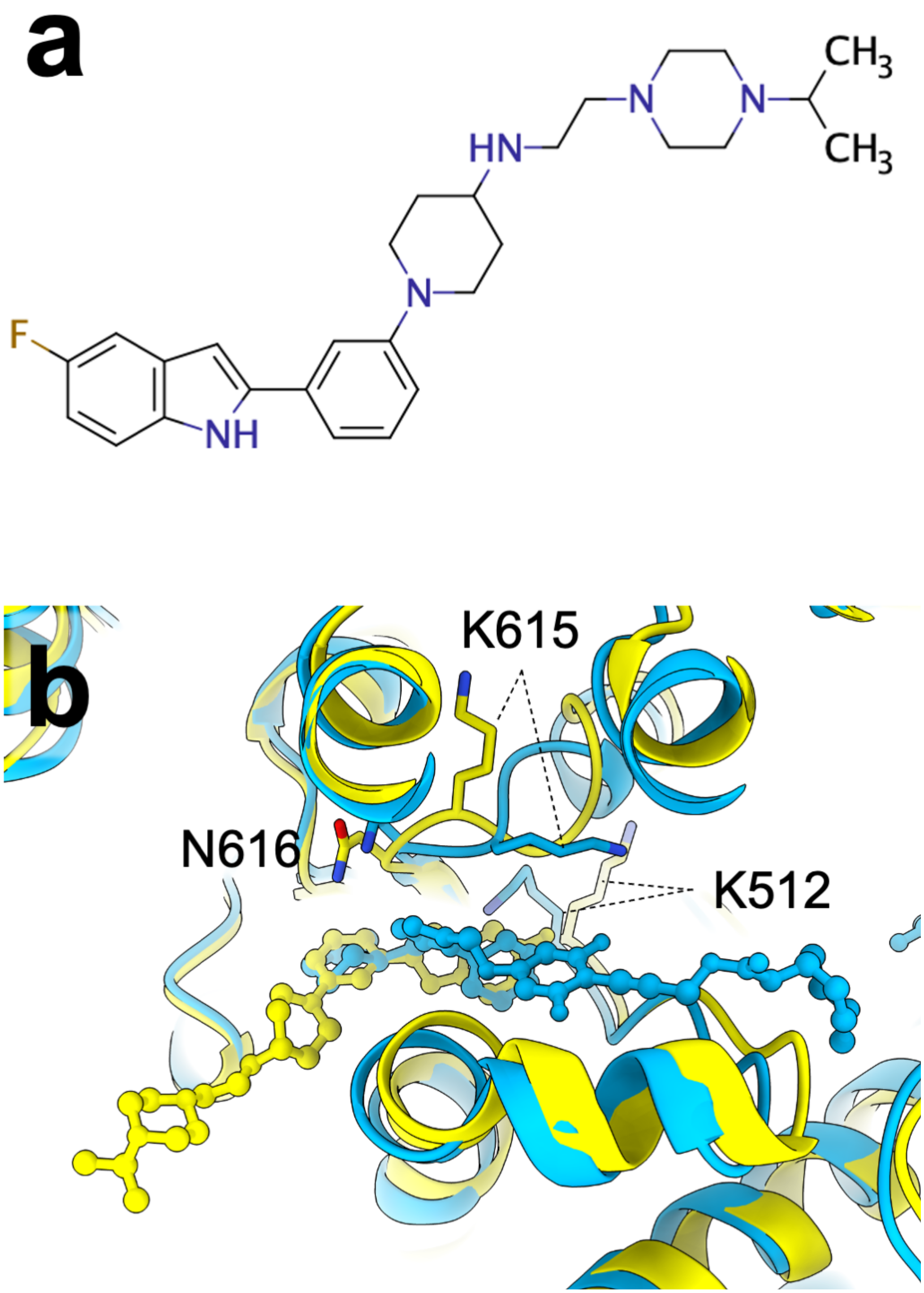

Supplementary Fig. 4 | UPCDC30245 binding site in p97 ATPase. a, Structure of UPCDC30245. **b,** Superposition of the allosteric inhibitors of triazole and phenyl indole. Structures of p97 with bounded NSC799462 and UPCDC30245 (PDB code: 5FTJ) (Banerjee et al., 2016) are shown in light blue and yellow, respectively. NSC799462 and UPCDC30245 are shown in ball-and-stick representation.

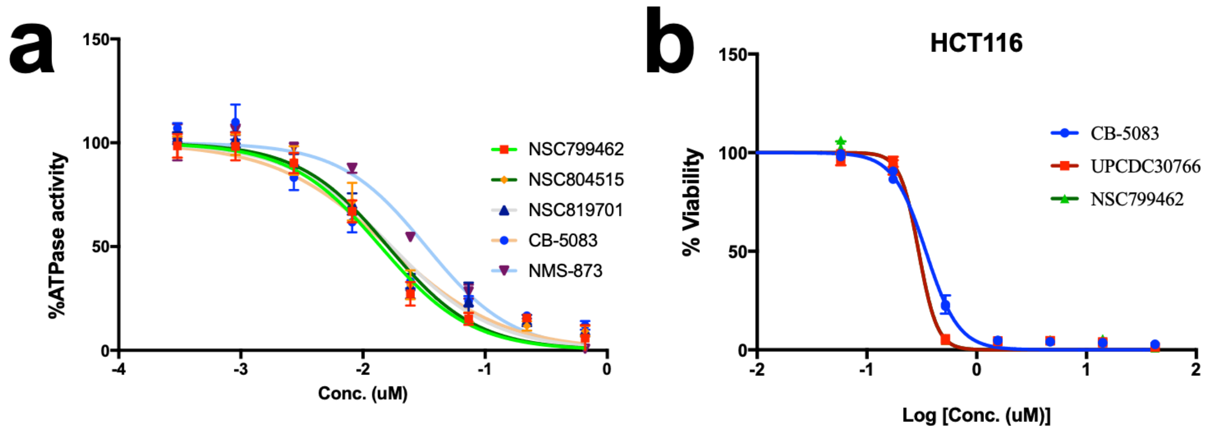

Supplementary Fig. 5 | Functional measurements of p97 ATPase activity and cellular viability impacted by p97 inhibitors. **a,** Titration curves of $p97^{WT}$ ATPase activity versus individual inhibitor concentration. Concentrations are in a logarithmic scale. **b,** Cellular viability of HCT116 when treated with p97 inhibitors.

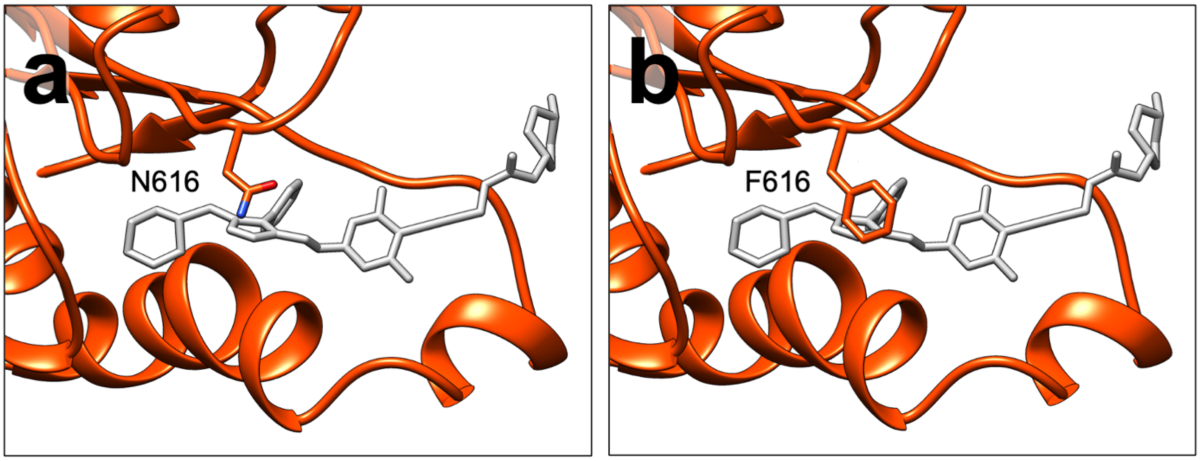

Supplementary Fig. 6 | *In silico* mutation of the residue 616 from asparagine to phenylalanine of p97 in the presence of NSC799462. Orange-red (cartoon) and white (stick) are p97 D2 ATPase domain and NSC799462, respectively.

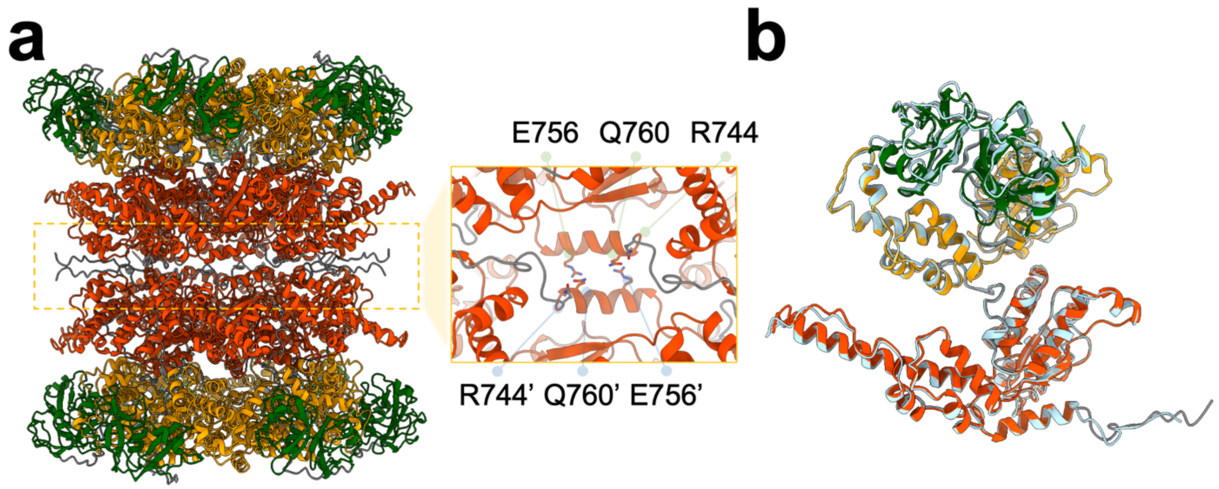

Supplementary Fig. 7 | Dodecameric p97|$_{NSC799462}$ assembly. **a,** Atomic model of the dodecameric p97|$_{NSC799462}$ assembly. N-, D1, D2 domains and linkers are colored in dark green, orange, orange-red, and grey. Right panel is the enlarged view of the interface between the two opposite D2 rings. Interacting residues are labelled. **b,** Superposition of the two p97|$_{NSC799462}$ monomers from a hexamer and a dodecamer (RMSD 0.778 Å). The p97|$_{NSC799462}$ monomer from the hexamer is colored in light blue.

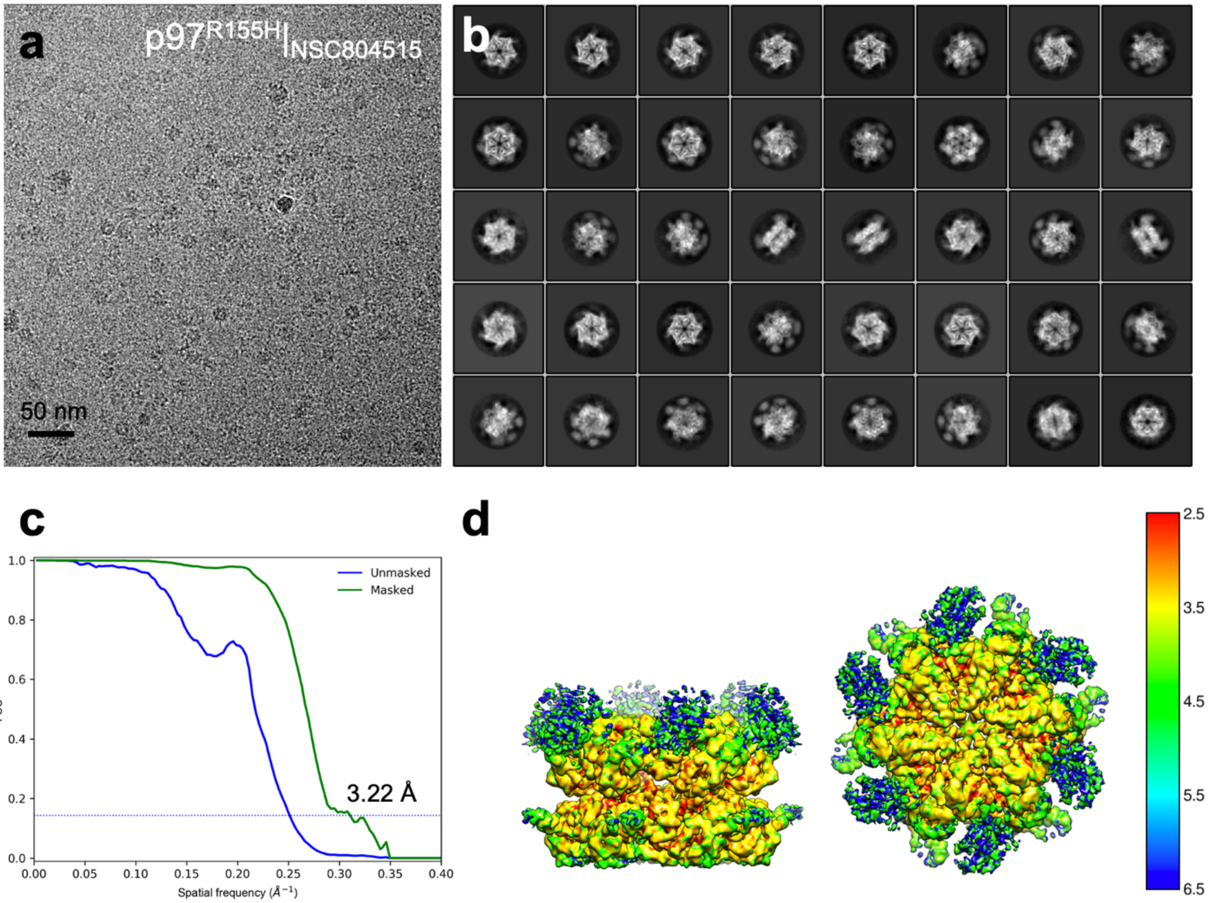

Supplementary Fig. 8 | Single-particle cryo-EM analysis of p97^{R155H} with the lead compound NSC804515. **a,** Electron micrograph of the p97^{R155H} with NSC804515. Scale bar indicates 50 nm. **b,** Representative two-dimensional (2D) class averages of single-particle images of p97^{R155H} with NSC804515. Box side length is 30.9 nm. **c,** Fourier shell correlation (FSC) plots of the two three-dimensional (3D) reconstructions. The resolutions were determined using gold standard FSC criteria at 0.143 cutoff. **d,** Local resolution estimate of the 3D reconstructions.

$p97^{R155H}|_{NSC804515}$

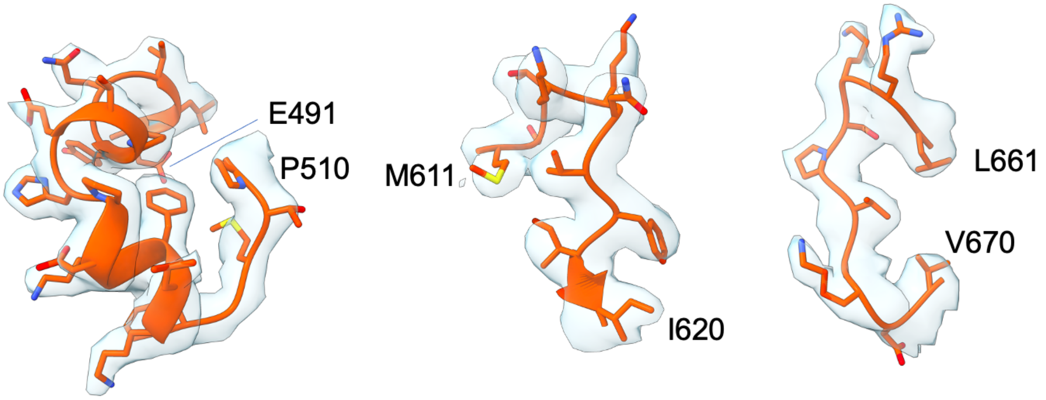

Supplementary Fig. 9 | Model fitting of the cryo-EM density map of $p97^{R155H}|_{NSC804515}$. Light blue surfaces represent cryo-EM densities.

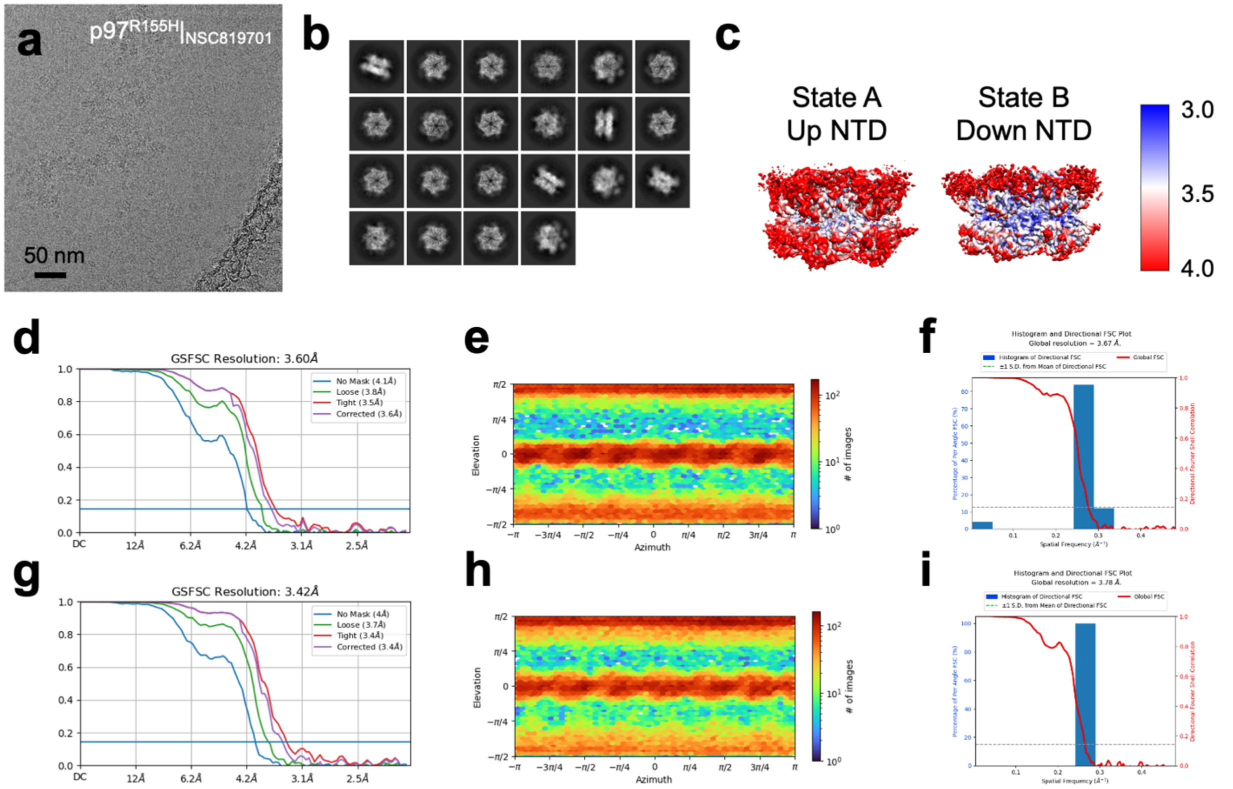

Supplementary Fig. 10 | Single-particle cryo-EM analysis of p97^{R155H} with the lead compound NSC819701. a, Electron micrograph of the cryogenic p97^{R155H}|$_{NSC819701}$. Scale bar indicates 50 nm. **b,** Representative two-dimensional (2D) class averages of single-particle images of p97^{R155H}|$_{NSC819701}$. Box side length is 36.9 nm. **c,** Local resolution map of the three-dimensional (3D) reconstructions of up-NTD (left; State A) and down-NTD (right; State B) p97^{R155H}|$_{NSC819701}$, respectively. **d** and **g,** Fourier shell correlation (FSC) plots of the two 3D reconstructions. Upper and lower plots are for up-NTD and down-NTD p97^{R155H}|$_{NSC819701}$, respectively. The resolutions were determined using gold standard FSC criteria at 0.143 cutoff. **e** and **h,** Euler angle distribution of the particle images for the reconstructions of the p97^{R155H}|$_{NSC819701}$ in State A and B, respectively. **f** and **i**, 3DFSC plots of the reconstructions of the p97^{R155H}|$_{NSC819701}$ in State A and B, respectively.

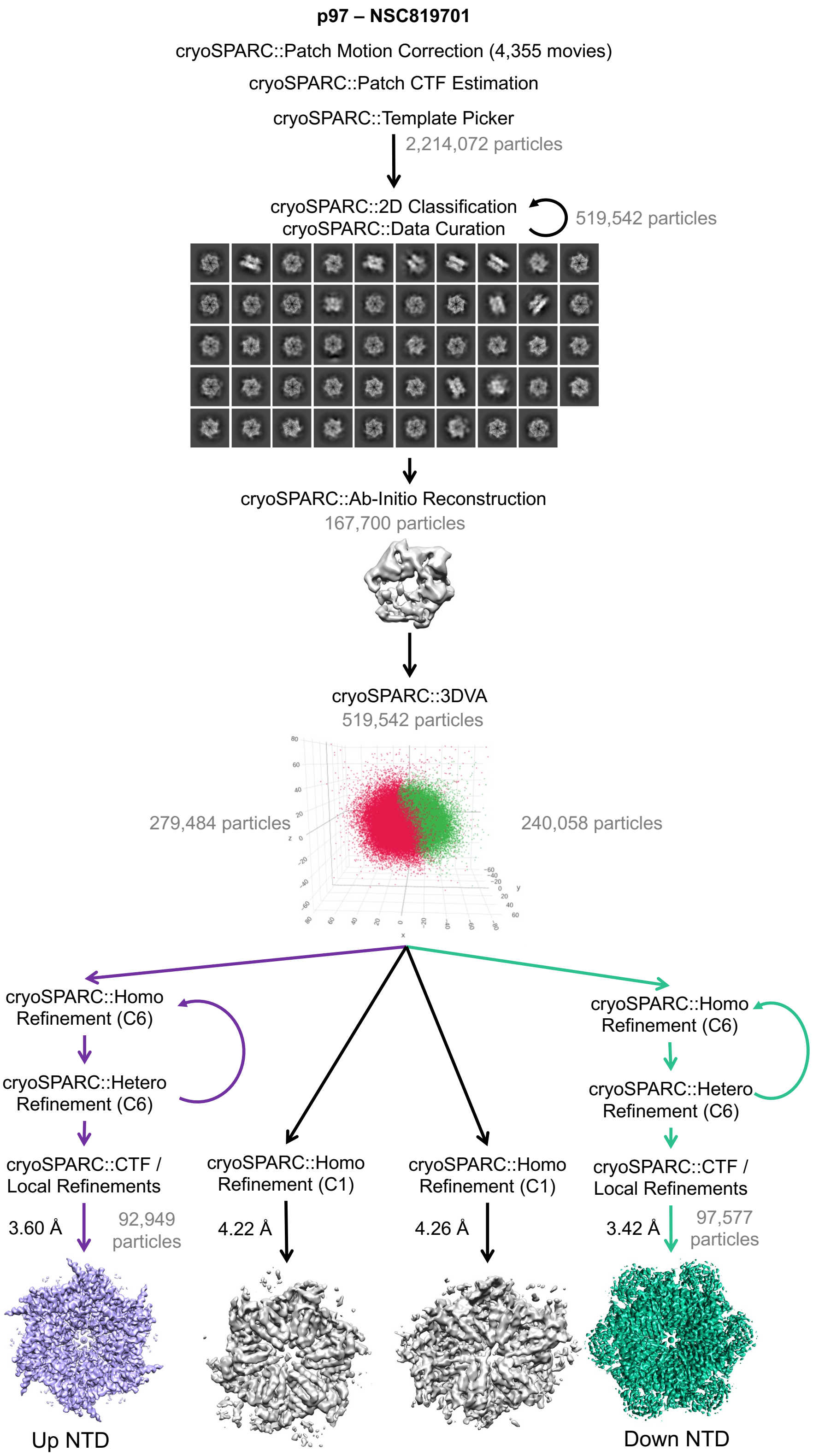

Supplementary Fig. 11 | Flowchart of single-particle cryo-EM image analysis of p97^{R155H}|$_{NSC819701}$.

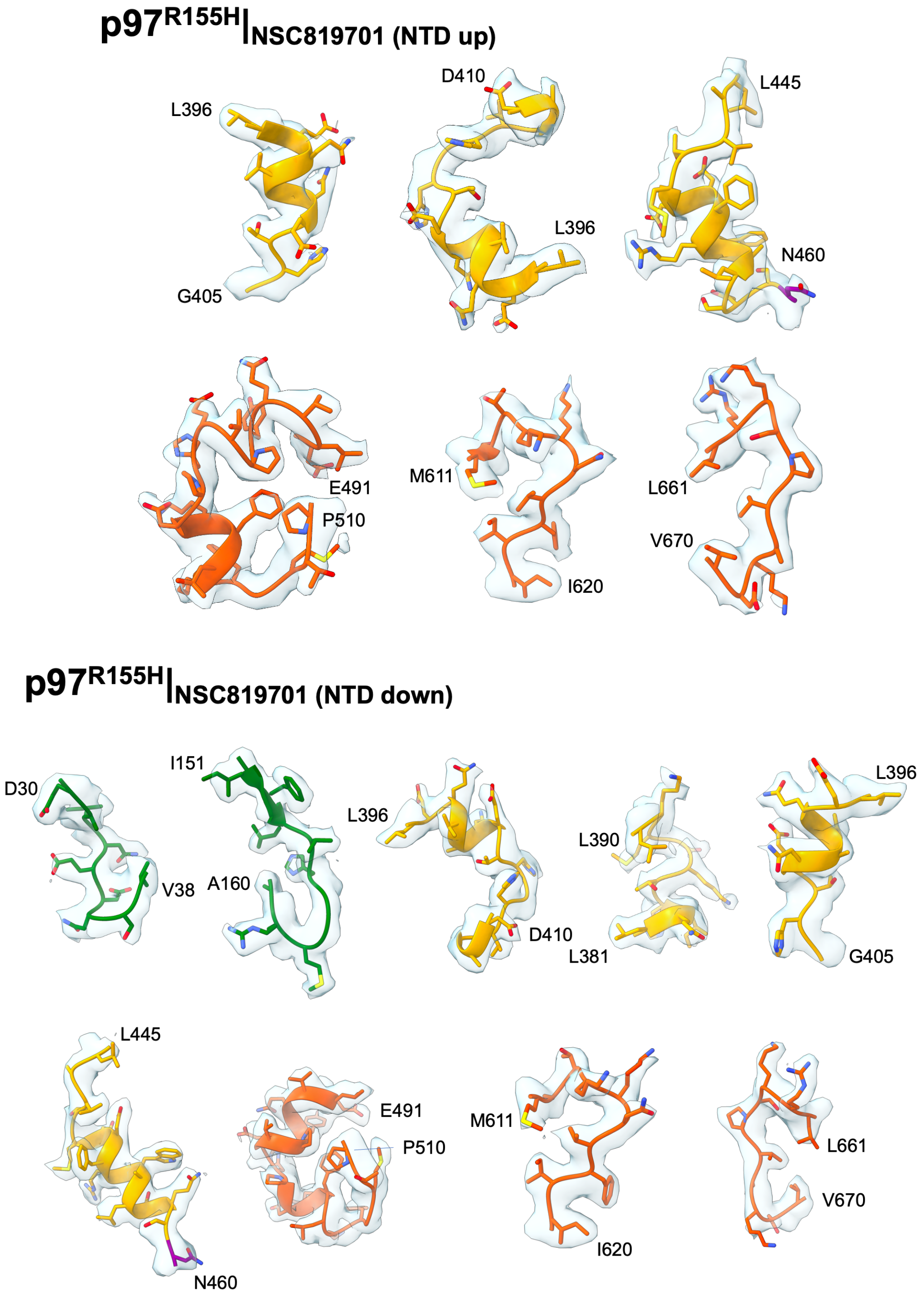

Supplementary Fig. 12 | Model fitting of the cryo-EM density map of p97^{R155H}|$_{NSC819701}$. Light blue surfaces represent cryo-EM densities.

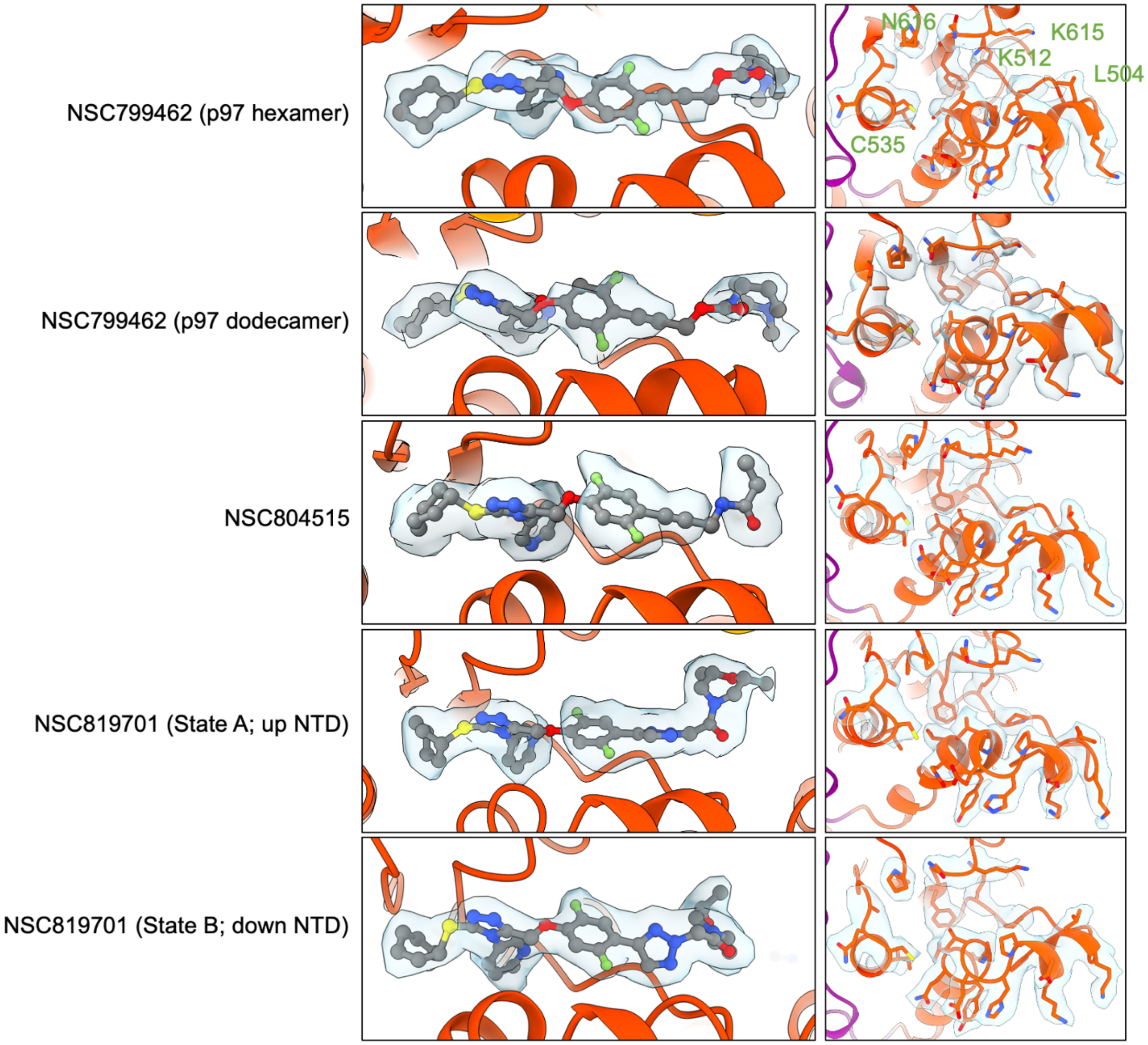

Supplementary Fig. 13 | Cryo-EM densities of the triazole compounds used in this study and bound residues of p97 ATPase. Orange-red cartoon is p97 D2 domain. Triazole compounds are presented in sticks. Yellow, light green, blue, and red ball representations are for sulfur, fluorine, nitrogen, and oxygen atoms. Cryo-EM densities are presented in light blue surfaces.

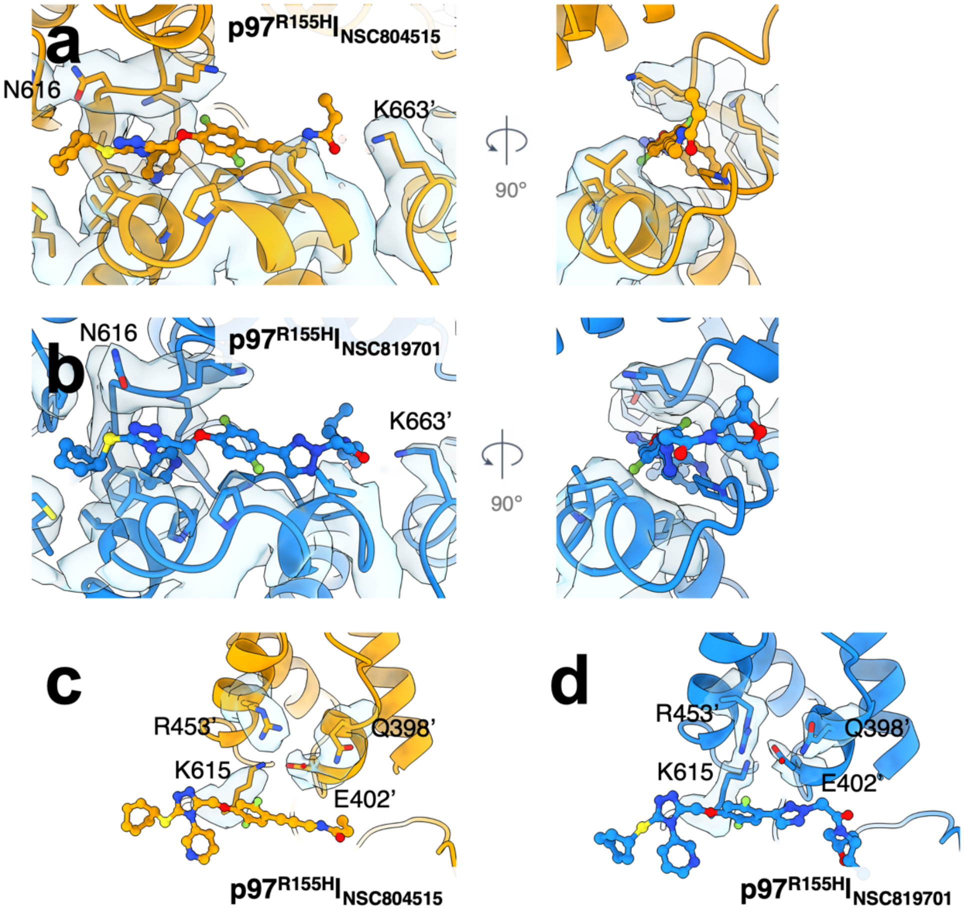

Supplementary Fig. 14 | Model-map density fitting of the structural regions shown in Fig. 5. **a** and **b,** Density fitting of the area mentioned in **Fig. 5a**. **c** and **d**, Density fitting of the area mentioned in **Fig. 5c**.

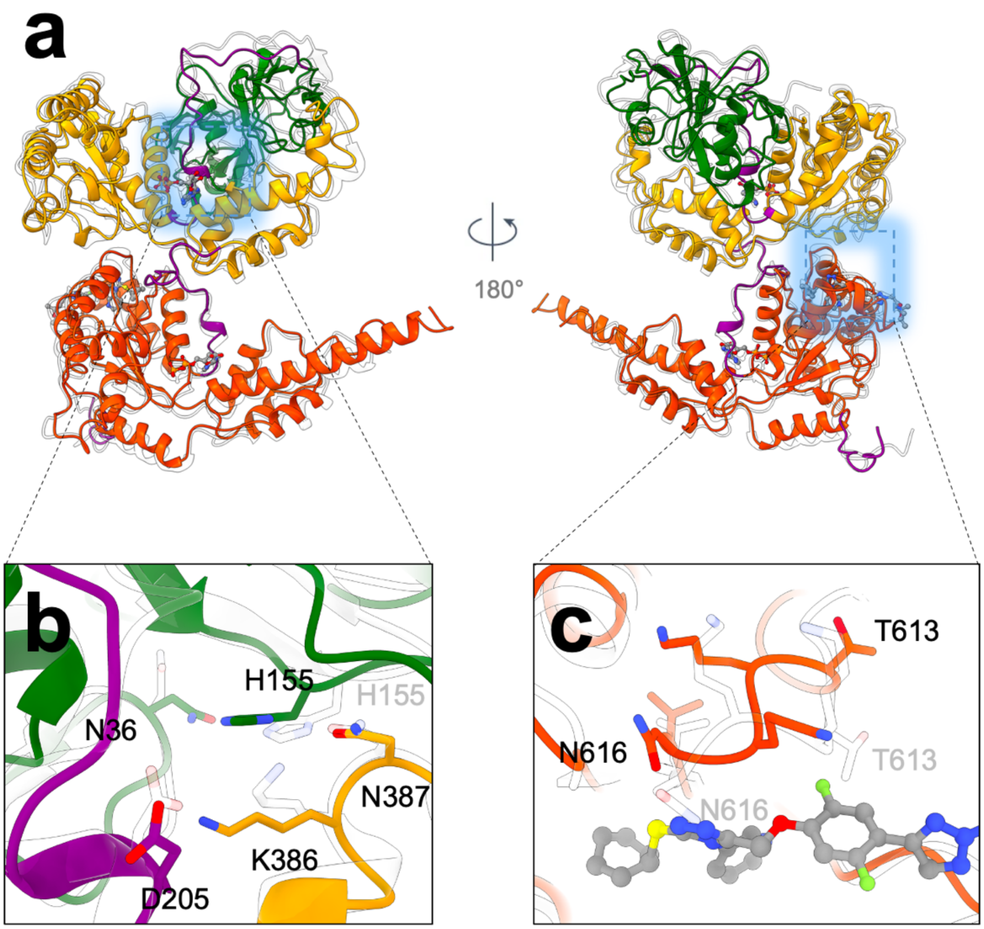

Supplementary Fig. 15 | Structure superposition of p97^{R155H}|$_{NSC819701}$ (State B; down NTD) and p97^{R155H} (PDB code: 7RL6) (RMSD 1.151 Å). Green, orange, orange red, and purple are NTD, D1, D2, and linker domains, respectively. Nucleotides and inhibitors are presented in sticks. **a,** Superposition of the two structures. p97^{R155H} structure is in white. **b,** Interactions of H155 with the surrounding residues. **c,** Loop structure of T613-V617 impacted by NSC819701 binding.

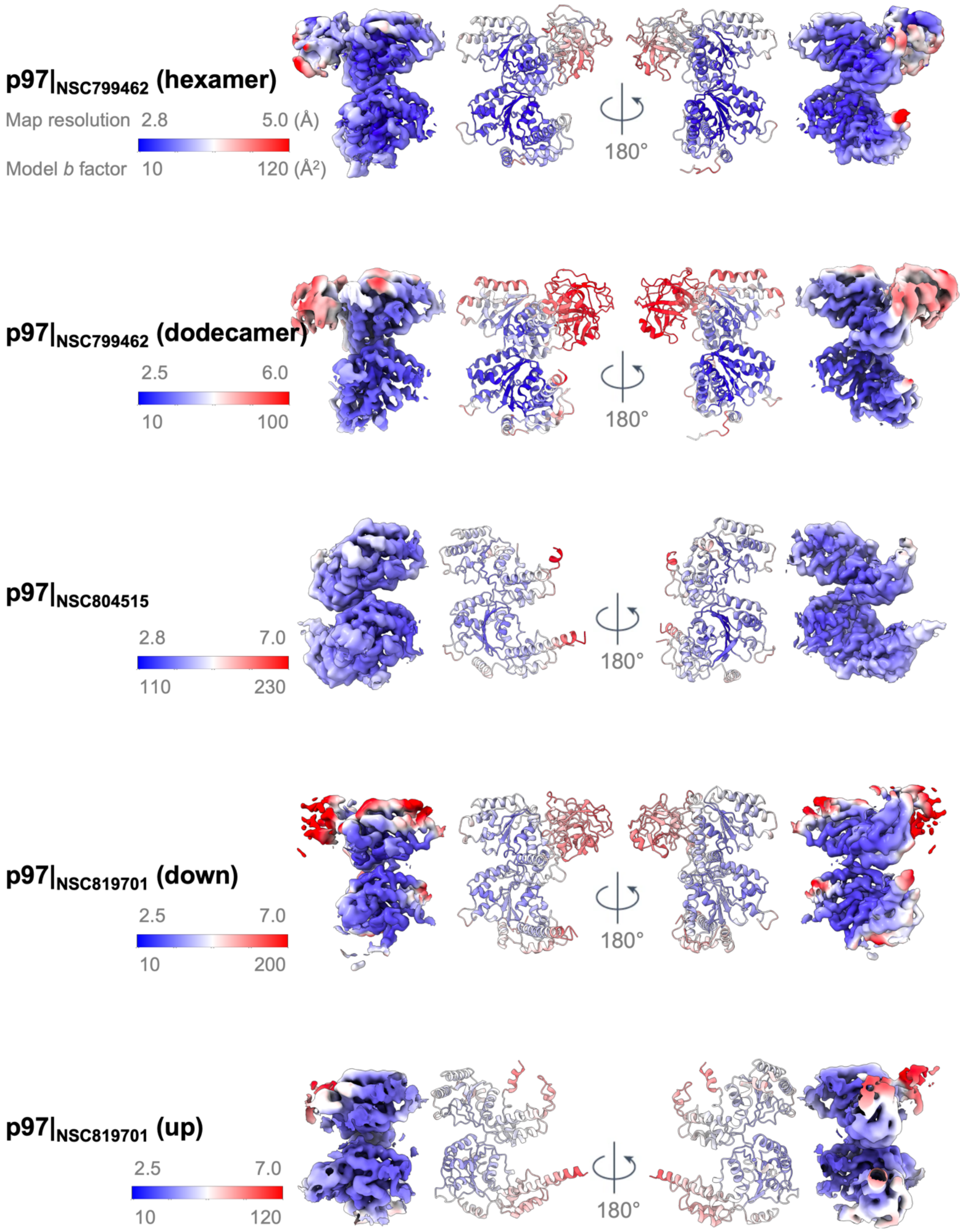

Supplementary Fig. 16 | Local resolution map estimations and model *b* factor distributions of the p97 ATPase with triazole inhibitors. Map local resolution estimation and model *b* factors of the studied cryo-EM densities are shown from blue (high resolution or low *b* factor value) to red (low resolution or high *b* factor value).

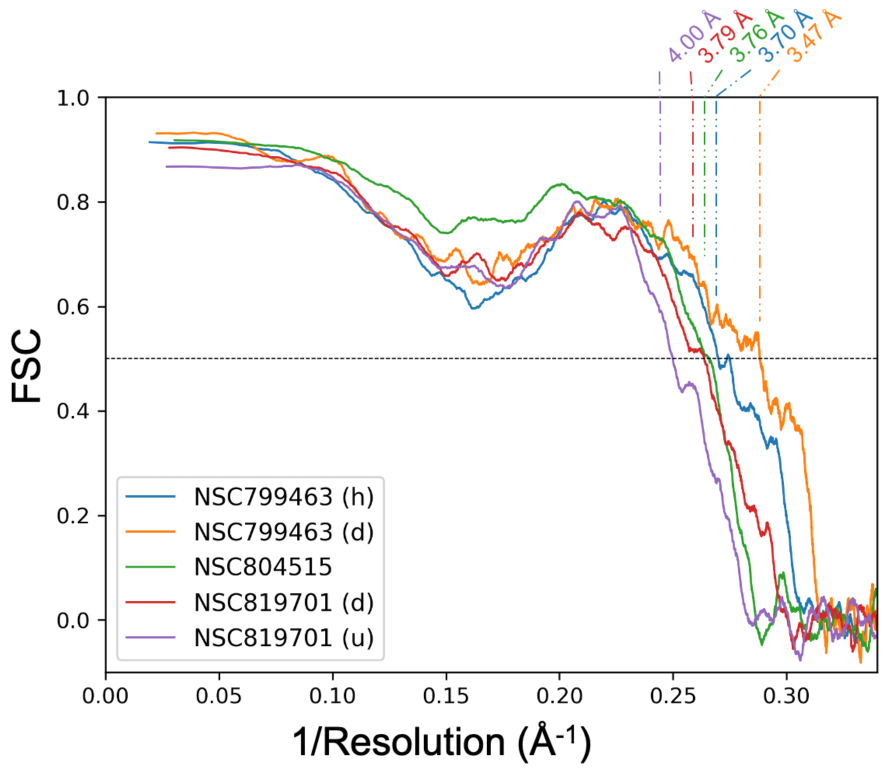

Supplementary Fig. 17 | Model-map Fourier shell correlation (FSC) plot of cryo-EM modeling.

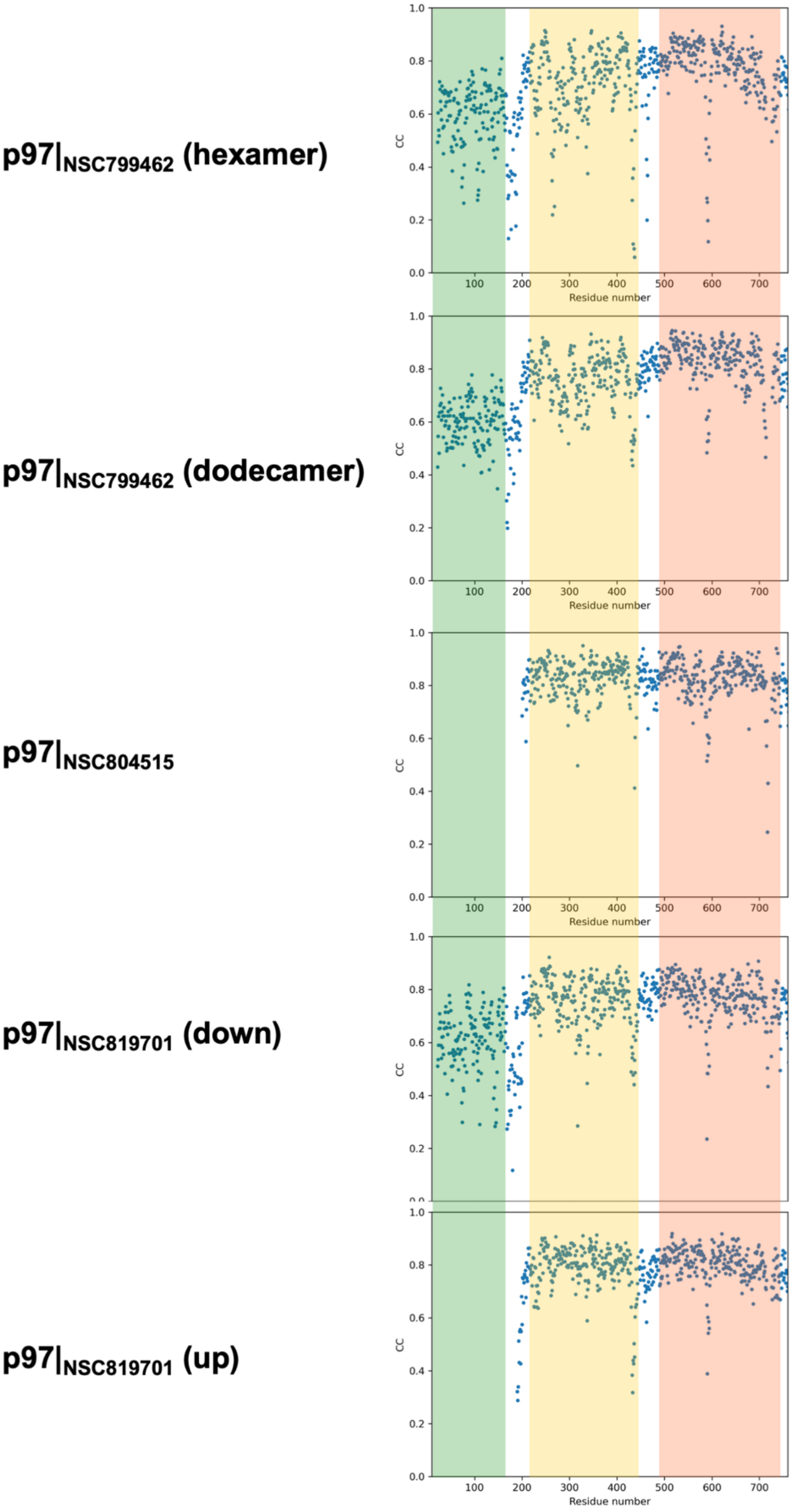

Supplementary Fig. 18 | Cross-correlation between the model and map per residue of cryo-EM modeling. Green, orange, and orange red are NTD, D1, and D2 domain residues, respectively.

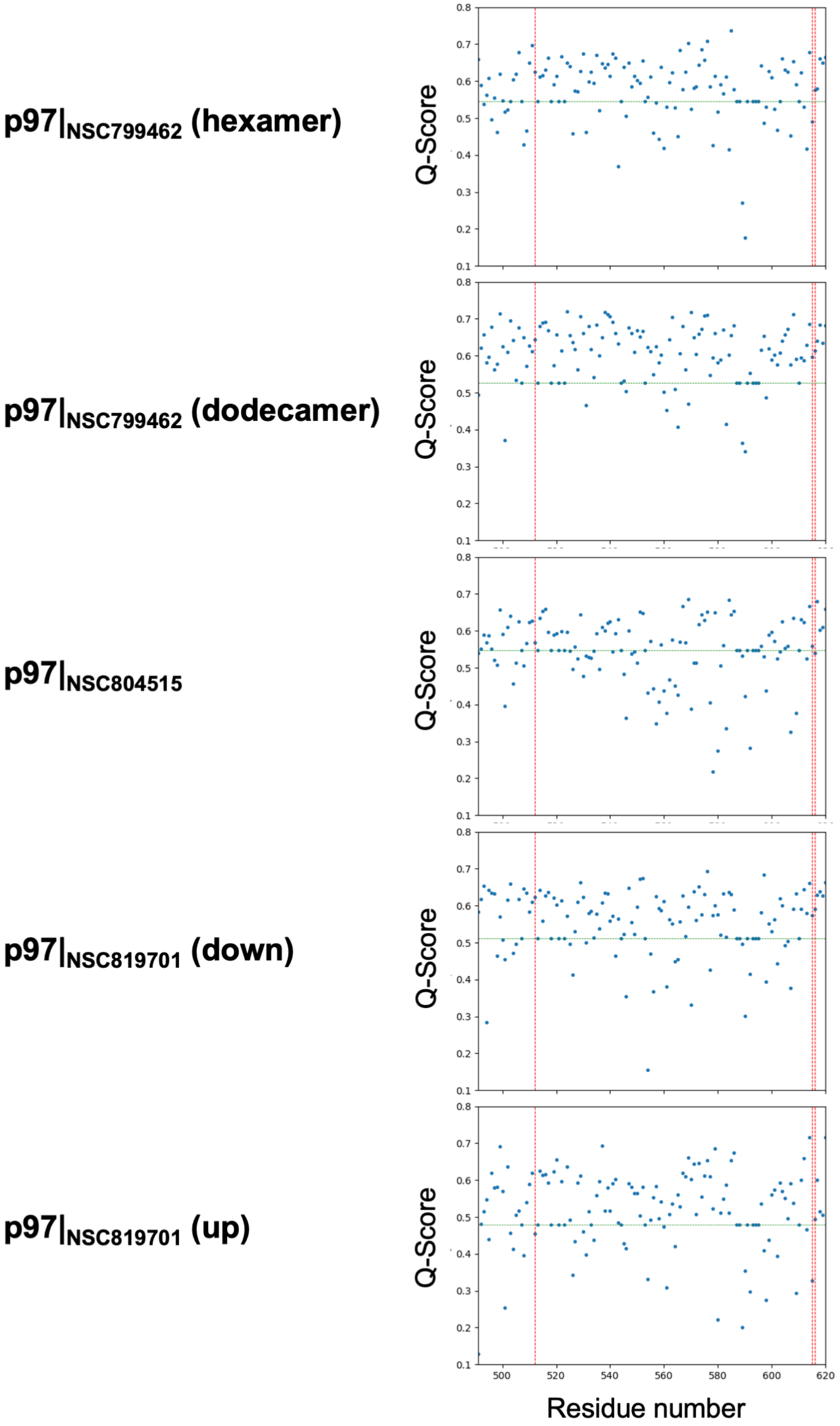

Supplementary Fig. 19 | Q-score estimation on the residues surrounding the inhibitor binding site. Red vertical lines indicate the key residues K512, K615, and N616, and green horizontal lines are the expected Q-scores at specific resolutions determined.

SUPPLEMENTARY TABLES

Supplementary Table S1. Plasmids used in this study.

Plasmid Number	Plasmid Name	Vector	Source and Reference
TCB-197	Human p97 pET15_T	pET15b_TEV linker	Chou, 2014 [1]
TCB-210	Human R155H p97 pET15_T	pET15b_TEV linker	Chou, 2014 [1]
TCB-515	Human K512N p97 pET15_T	pET15b_TEV linker	This study
TCB-573	Human P510S p97 pET15_T	pET15b_TEV linker	This study
TCB-529	Human N616F p97 pET15_T	pET15b_TEV linker	This study
TCB-576	Human F618S p97 pET15_T	pET15b_TEV linker	This study
TCB-592	Human D649A/T688A p97 pET15_T	pET15b_TEV linker	This study

1] T. F. Chou, S. L. Bulfer, C. C. Weihl, K. Li, L. G. Lis, M. A. Walters, F. J. Schoenen, H. J. Lin, R. J. Deshaies, M. R. Arkin, *J. Mol. Biol.* **2014**, *426*(15), 2886-2899.

Supplementary Table S2. Primers used in this study.

Name of Primer	Target of Primer	Sequence of Primer
PCR Primer 1	VCP-Cloning F	5'- CAG CGT TGT TCG CCC -3'
PCR Primer 2	VCP-Cloning R	5'- ACC CCC AGG GAA CAA G -3'
Sequencing Primer 1	VCP-Cloning F	5'- CAG CGT TGT TCG CCC -3'
Sequencing Primer 2	p97seq301F	5'- GCA TCC AGC CAT GCC CTG ATG TG -3'
Sequencing Primer 3	p97bp803F	5'- GAC CCT GAT TGC TCG AGC TG -3'
Sequencing Primer 4	p97 b1189F	5'- GAA CAG GTA GCC AAT GAG ACT -3'
Sequencing Primer 5	Hp97 1501-F	5'- GAC AAA TTC CTG AAG TTT GGC -3'
Sequencing Primer 6	p97seq1976F	5'- CTA ACC TGC GCA AGT CCC CAG TTG -3'

Supplementary Table S3. EC_{50} values (μM) for p97 Ub^{G76V}-GFP reporter and cellular viability assay.

IC_{50} (μM) *	Ub^{G76V}-GFP EC_{50} (μM)	HCT116 $p97^{WT}$	HCT116 $p97^{P510S}$	HCT116 $p97^{K512N}$	HCT116 $p97^{F618S}$
**NMS-873**	2.0	2.0	9.2	ND	ND
**UPCDC30766**	0.27	0.29	10.3	7.3	ND
**UPCDC30732 (racemic mixture of UPCDC30766)**	0.45	0.76	10.6	15	8
**UPCDC30733 (racemic mixture of NSC799462)**	0.53	0.68	9.6	25	21
**NSC799462**	0.23	0.39	6.9	ND	ND
**NSC804515**	0.87	0.75	ND	ND	ND
**NSC819701**	0.66	0.83	ND	ND	ND
**CB-5083**	0.43	0.40	1	0.5	0.6

* Measurements were carried out in four replicates.

ND = 'Not determined'

Supplementary Table S4. IC_{50} values (μM) for p97 inhibitors against the wild-type and mutant p97 at 200 μM ATP.

IC_{50} (μM)*	**WT**	**R155H**	**P510S**	**K512N**	**N616F**	**F618S**
**NMS-873**	0.032	0.033	2.14	0.28	> 6.6	3.39
Ratio**	1	1	67	8.8	> 206	106
**NSC799462**	0.015	0.011	0.91	0.045	1.81	0.46
Ratio	1	0.7	61	3	121	31
**NSC804515**	0.015	0.014	2.32	0.18	> 6.6	1.75
Ratio	1	0.9	155	12	> 440	117
**NSC819701**	0.016	0.015	2.80	0.12	5.34	1.51
Ratio	1	0.9	175	7.5	334	94
**CB-5083**	0.016	0.020	0.013	0.013	0.019	0.015
Ratio	1	1.3	0.8	0.8	1.2	0.9
**UPCDC30766**	0.012	ND	ND	0.07	> 0.66	ND
Ratio	1			6	> 56	

* Measurements were carried out in six replicates.

** Ratio of IC_{50} values for mutants to IC_{50} values for wild-type p97.

ND = 'Not determined'

Supplementary Table S5. Assessment of p97 ATPase activity through titration with varying concentrations of individual inhibitors.

Concentration (μM) / % ATPase activity	**NMS-873**	**NSC799462**	**NSC804515**	**NSC819701**	**CB-5083**
**0**	100	99	98	102	107
**0.000905**	106	98	99	102	110
**0.002716**	98	90	92	90	83
**0.008148**	88	67	72	71	62
**0.024444**	55	27	32	31	33
**0.073333**	28	15	16	23	24
**0.220000**	14	16	12	14	17
**0.660000**	1	6	9	9	12

Supplementary Table S6. Cell viability of HCT116 after 48-hour treatment with studied compounds that inhibit p97 ATPase activity.

	**IC_{50} (μM)**
**CB-5083**	0.344
**UPCDC30766**	0.296
**NSC799462**	0.296

Supplementary Table S7. Measurements of cellular viability of HCT116 treated with p97 inhibitors.

Concentration (μM)	**CB-5083**		**UPCDC30766**		**NSC799462**	
**0.00**	94	96	98	103	98	98
**0.06**	99	97	94	99	100	105
**0.06**	100	99	98	101	108	105
**0.17**	87	86	97	94	94	93
**0.17**	90	92	95	90	93	96
**0.52**	24	21	6	5	6	6
**0.52**	26	20	5	5	5	5
**1.60**	5	5	4	5	5	4
**1.60**	5	4	4	4	5	5
**4.70**	4	4	5	4	5	5
**4.70**	4	4	4	5	5	5
**14.0**	4	4	4	4	5	6
**14.0**	4	3	3	4	6	6
**42.1**	3	3	2	2	2	1
**42.1**	3	3	1	1	1	1

Supplementary Table S8. Single-particle cryo-EM data collection, refinement, and validation statistics of p97 ATPase with the allosteric inhibitors.

	$p97^{R155H}|_{NSC804515}$ (EMD-42625) (PDB code: 8UVO)	$p97^{R155H}|_{NSC819701}$		$p97|_{NSC799462}$	
		State A (up NTD) (EMD-42626) (PDB code: 8UVP)	State B (down NTD) (EMD-42627) (PDB code: 8UVQ)	Hexamer (EMD-42603) (PDB code: 8UV2)	Dodecamer (EMD-44748) (PDB code: 9BOQ)
**Data collection and processing**					
Magnification			48,077		
Voltage (kV)			300		
Electron exposure ($e^-/Å^2$)			47.84		
Defocus range (μm)			-0.8 - -2.5		
Pixel size (Å)			1.04		
Symmetry imposed	C6	C6	C6	C6	D6
Initial particle images	97,583	128,935		168,754	
Final particle images	27,998	31,543	42,197	22,491	100,402
Map resolution (Å)	3.22	3.60	3.42	3.23	3.33
FSC threshold at 0.143					
**Refinement**					
Initial model used (PDB code)	5FTN	5FTN	5FTK	5FTK	5FTK
Model resolution (Å)					
FSC threshold at 0.5	3.76	4.00	3.79	3.70	3.47
Map sharpening *b* factor ($Å^2$)	-79.7	-143.3	-140.1	-117.0	-105.7
Model composition					
Non-hydrogen atoms	26,202	26,556	35,604	35,580	70,572
Protein residues	3,294	3,330	4,476	4,458	8,832
Ligands	18	18	18	18	36
ADP	12	12	12	12	24
Triazole inhibitor	6	6	6	6	12
B factors ($Å^2$)					
Protein	144.3	78.14	83.41	50.57	59.60
Ligand	137.4	66.76	45.64	38.22	28.72
RMS deviations					
Bond lengths (Å)	0.003	0.002	0.003	0.004	0.002
Bond angles (°)	0.673	0.622	0.635	0.739	0.646
Validation					
MolProbity score	1.82	1.98	2.00	1.91	2.05
Clashscore	6.46	6.29	9.04	7.30	11.45
Poor rotamers (%)	0.00	0.00	0.00	0.26	0.11
Ramachandran plot					
Favored (%)	92.66	86.21	91.19	91.32	92.19
Allowed (%)	7.34	13.43	8.81	8.68	7.66
Disallowed (%)	0.00	0.36	0.00	0.00	0.15
