# Supplementary material for: Mechanism of allosteric inhibition of human p97/VCP ATPase and its disease mutant by triazole inhibitors
Source: Commun Chem. 2024 Aug 9;7:177. doi: 10.1038/s42004-024-01267-3 (PMC11316111; doi:10.1038/s42004-024-01267-3)
Supplement: Supplementary file 3 — Description of Additional Supplementary Files [file 42004_2024_1267_MOESM3_ESM.pdf]

Description of Additional Supplementary Files

File name: Supplementary Data 1 - 3

Description: NMR spectroscopy data of the triazole allosteric inhibitors used in this study.

File name: Supplementary Movie 1

Description: Structural morphing of p97 ATPase impacted by NSC799463 binding.

File name: Supplementary Movie 2

Description: Structural morphing of NSC-bound p97 R155H disease mutant during up and down NTD transitions.
