# Supplementary material for: Mechanism of allosteric inhibition of human p97/VCP ATPase and its disease mutant by triazole inhibitors
Source: Commun Chem. 2024 Aug 9;7:177. doi: 10.1038/s42004-024-01267-3 (PMC11316111; doi:10.1038/s42004-024-01267-3)
Supplement: Supplementary file 4 — Supplementary Data 1–3 [file 42004_2024_1267_MOESM4_ESM.pdf]

SUPPLEMENTARY DATA

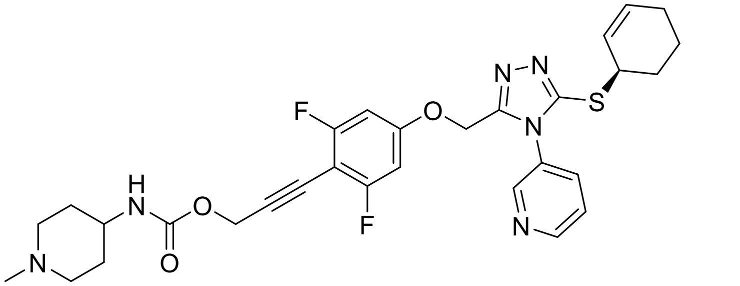

(*R*)-3-(4-((5-(cyclohex-2-en-1-ylthio)-4-(pyridin-3-yl)-4H-1,2,4-triazol-3-yl)methoxy)-2,6-difluorophenyl)prop-2-yn-1-yl(1-methylpiperidin-4-yl)carbamate

NMR spectroscopy data:

$[\alpha]_D$ = +72.2 (*c* 0.175, Methanol); ^{1}H NMR (500 MHz, DMSO-d_6) δ 8.73 (dd, J = 5.0, 1.5 Hz, 1H), 8.70 (d, J = 2.0 Hz, 1H), 8.01–7.99 (m, 1H), 7.63–7.60 (m, 1H), 7.37 (d, J = 7.5 Hz, 1H), 6.84 (d, J = 9.0 Hz, 2H), 5.88–5.84 (m, 1H), 5.68–5.65 (m, 1H), 5.25 (s, 2H), 4.89 (s, 2H), 4.30–4.29 (m, 1H), 3.32 (s, 1H), 2.69–2.67 (m, 2H), 2.12 (s, 3H), 1.99–1.82 (m, 6H), 1.70–1.68 (m, 2H), 1.62–1.56 (m, 2H), 1.44–1.36 (m, 2H); MS (ESI$^+$) *m/z* $[M+H]^+$ 595.

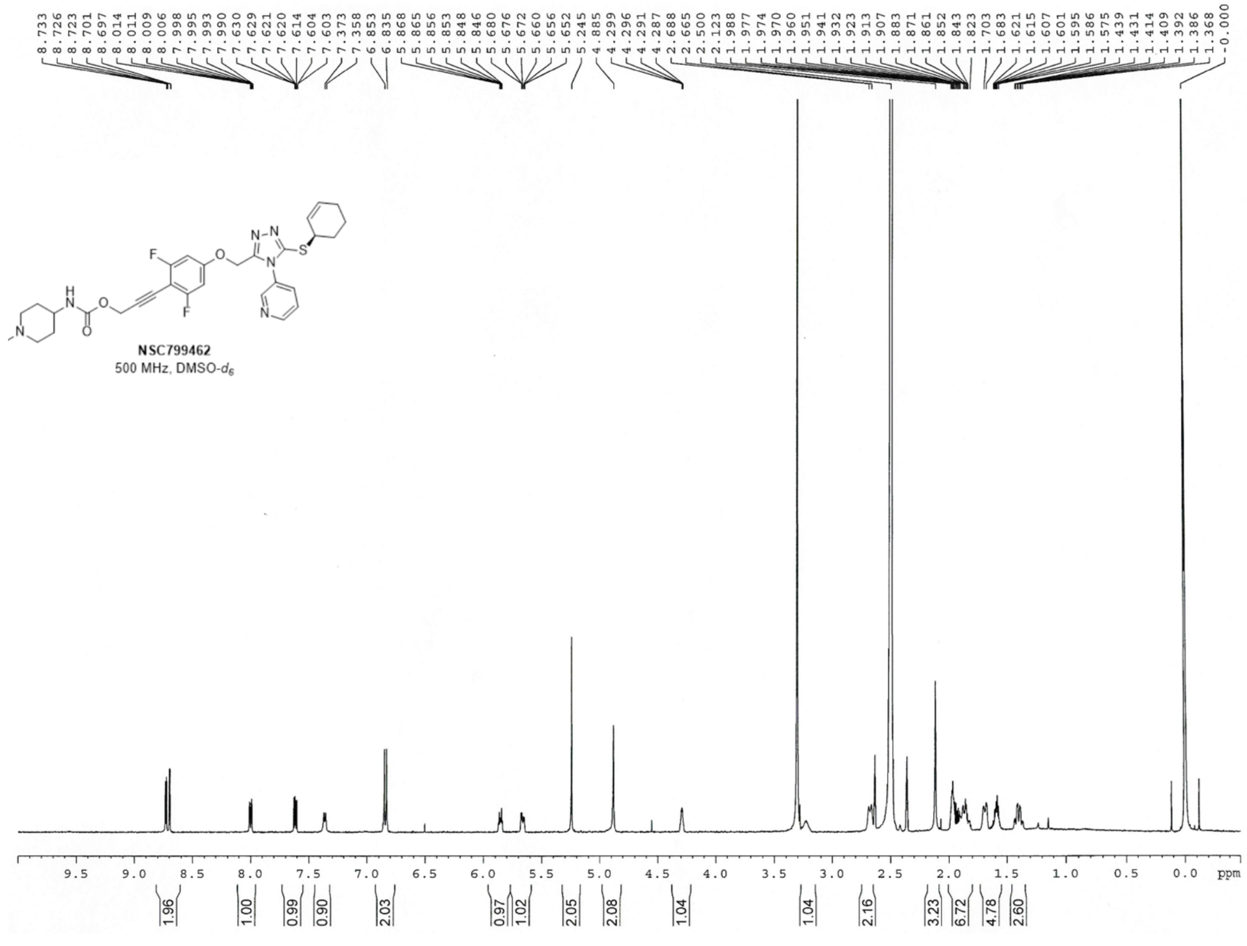

Supplementary Data 1 | Characterization of the triazole compound NSC799462.

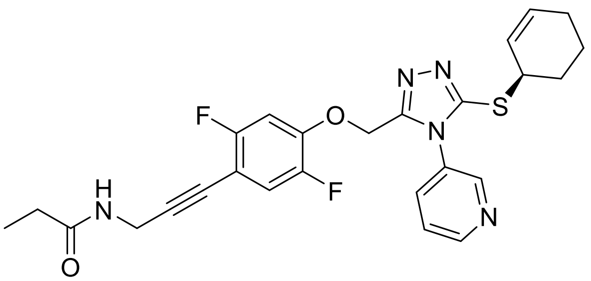

(*R*)-N-(3-(4-((5-(cyclohex-2-en-1-ylthio)-4-(pyridin-3-yl)-4H-1,2,4-triazol-3-yl)methoxy)-2,5-difluorophenyl)prop-2-yn-1-yl)propionamide

NMR spectroscopy data:
$[\alpha]_D$ = +122.7 (*c* 0.11, Methanol); ^{1}H NMR (300 MHz, DMSO-d_6) δ ^{1}H NMR (300 MHz, CDCl3) δ 8.73 (dd, J = 4.8, 1.5 Hz, 1H), 8.69 (d, J = 2.1 Hz, 1H), 8.32 (t, J = 5.3 Hz, 1H), 8.01–7.97 (m, 1H), 7.61 (dd, J = 8.4, 5.1 Hz, 1H), 7.39–7.29 (m, 2H), 5.87–5.84 (m, 1H), 5.68–5.64 (m, 1H), 5.32 (s, 2H), 4.29 (s, 1H), 4.11 (d, J = 5.4 Hz, 2H), 2.11 (q, J = 7.6 Hz, 2H), 1.96–1.81 (m, 4H), 1.62–1.55 (m, 2H), 0.99 (t, J = 7.5 Hz, 3H); MS (ESI^{+}) *m/z* $[M+H]^{+}$ 510.

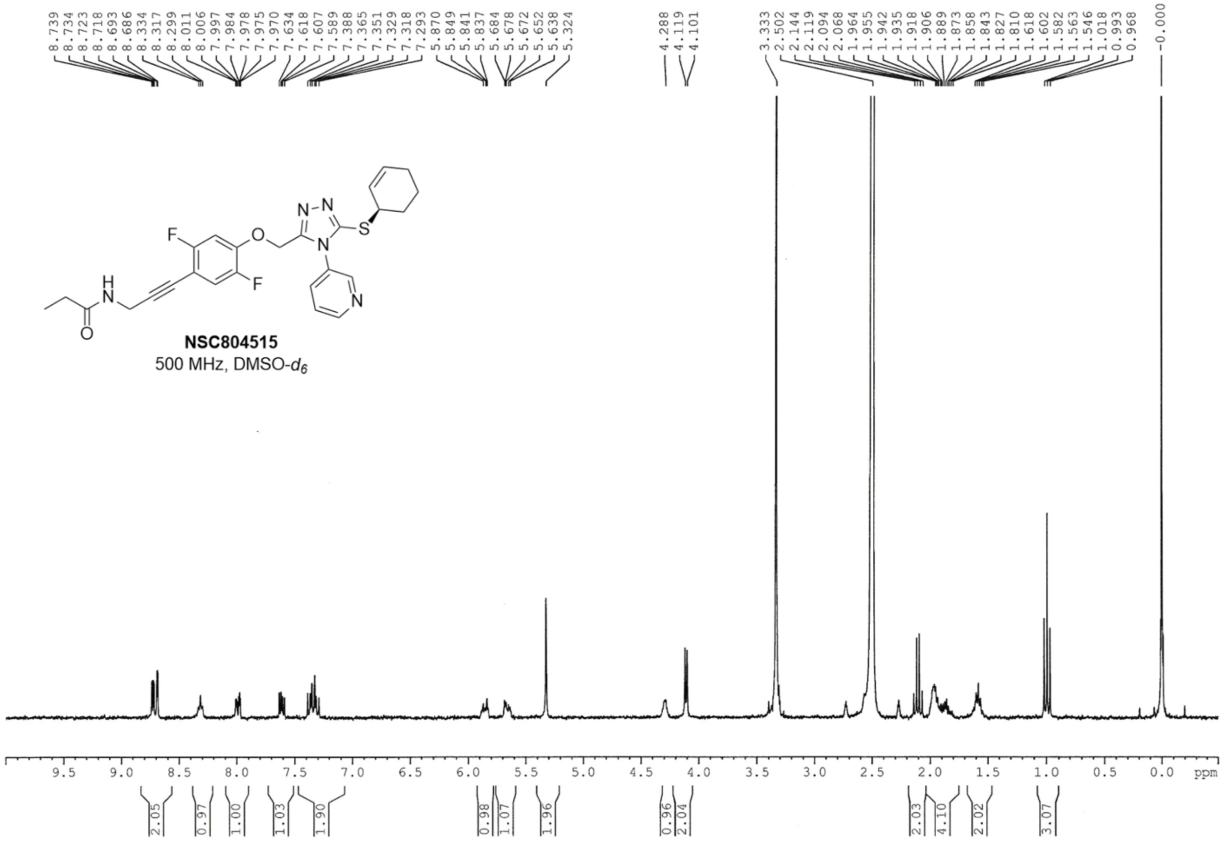

Supplementary Data 2 | Characterization of the triazole compound NSC804515.

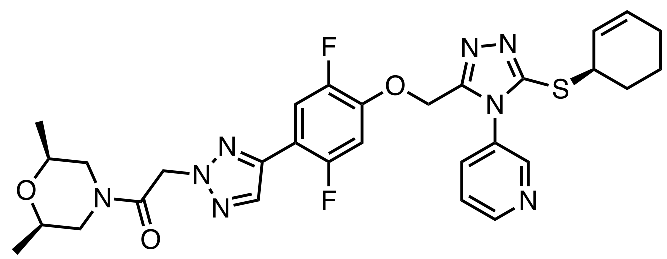

2-(4-(4-((5-(((*R*)-Cyclohex-2-en-1-yl)thio)-4-(pyridin-3-yl)-4*H*-1,2,4-triazol-3-yl)methoxy)-2,5-difluorophenyl)-2*H*-1,2,3-triazol-2-yl)-1-((2*S*,6*R*)-2,6-dimethylmorpholino)ethan-1-one

NMR spectroscopy data:

Mp 95-97 ºC; $[\alpha]_D$ = +102.7 (*c* 0.064, DMSO); IR (neat) 2860, 1665, 1547, 1485, 1445, 1391, 1270, 1074, 997, 847, 827, 749, 706 cm^{-1}; ^{1}H NMR (500 MHz, CD_3OD) d 8.75 (dd, *J* = 1.5, 5.0 Hz, 1 H), 8.71 (d, *J* = 2.5 Hz, 1 H), 8.03-8.01 (m, 1 H), 7.99 (d, *J* = 3.9 Hz, 1 H), 7.69-7.65 (m, 2 H), 7.17 (dd, *J* = 6.9, 11.7 Hz, 1 H), 5.94-5.90 (m, 1 H), 5.72-5.69 (m, 1 H), 5.56 (q, J_{AB} = 16.3 Hz, 2 H), 5.30 (s, 2 H), 4.39-4.37 (m, 1 H), 4.31 (dt, *J* = 1.9, 13.2 Hz, 1 H), 3.86 (dt, *J* = 1.9, 13.0 Hz, 1 H), 3.68-3.64 (m, 1 H), 3.61-3.56 (m, 1 H), 2.91 (dd, *J* = 10.7, 13.2 Hz, 1 H), 2.44 (dd, *J* = 10.9, 13.0 Hz, 1 H), 2.08-2.03 (m, 3 H), 1.97-1.92 (m, 1 H), 1.79-1.71 (m, 1 H), 1.69-1.63 (m, 1 H), 1.22 (d, *J* = 6.3 Hz, 3 H), 1.19 (d, *J* = 6.2 Hz, 3 H); ^{13}C NMR (125 MHz, $CDCl_3$) d 163.5, 155.5 (d, J_{CF} = 245 Hz), 154.0, 151.4, 150.7 (d, J_{CF} = 225 Hz), 150.5, 147.9, 145.5 (d, J_{CF} = 24 Hz), 142.1, 135.0, 134.3 (J_{CF} = 12 Hz), 132.5, 129.8, 125.4 124.2, 115.1 (d, J_{CF} = 5 Hz), 114.9 (J_{CF} = 5 Hz), 104.1 (J_{CF} = 28 Hz), 71.8, 71.6, 61.3, 56.0, 50.8, 47.5, 44.1, 29.2, 24.8, 19.2, 18.7, 18.6; ^{19}F NMR (470 MHz, CD_3OD) d -118.3 (d, *J* = 18.8 Hz), -140.1 (d, *J* = 18.8 Hz); HRMS (ESI^+) *m/z* calculated for $C_{30}H_{33}N_8O_3F_2S$ $[M+H]^+$ 623.2364, found 623.2324.

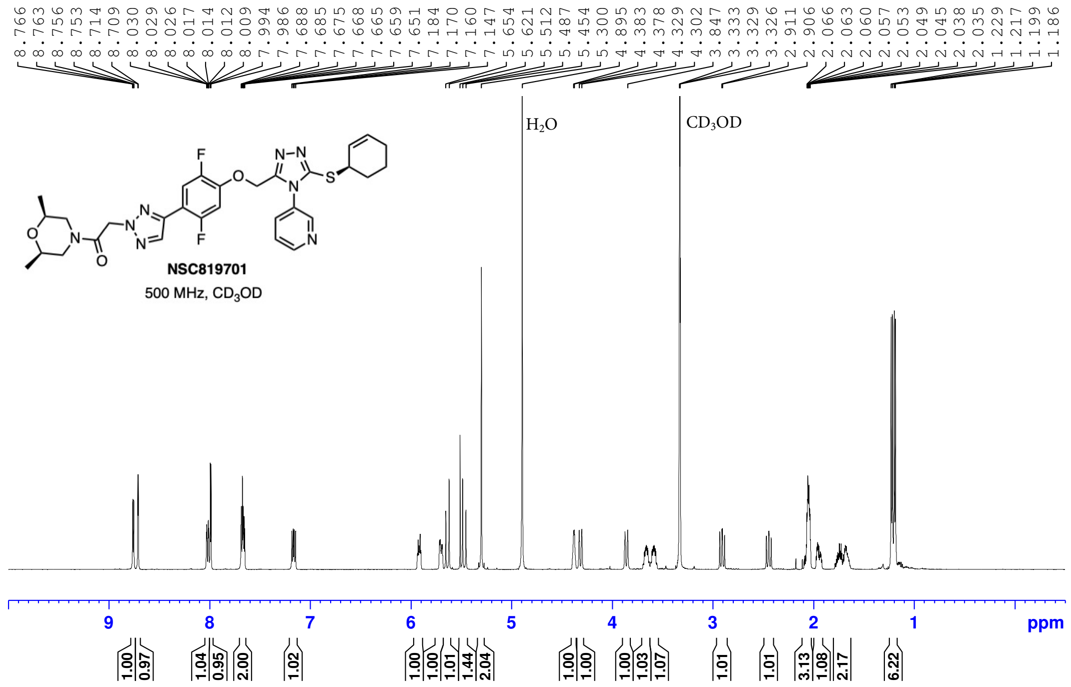

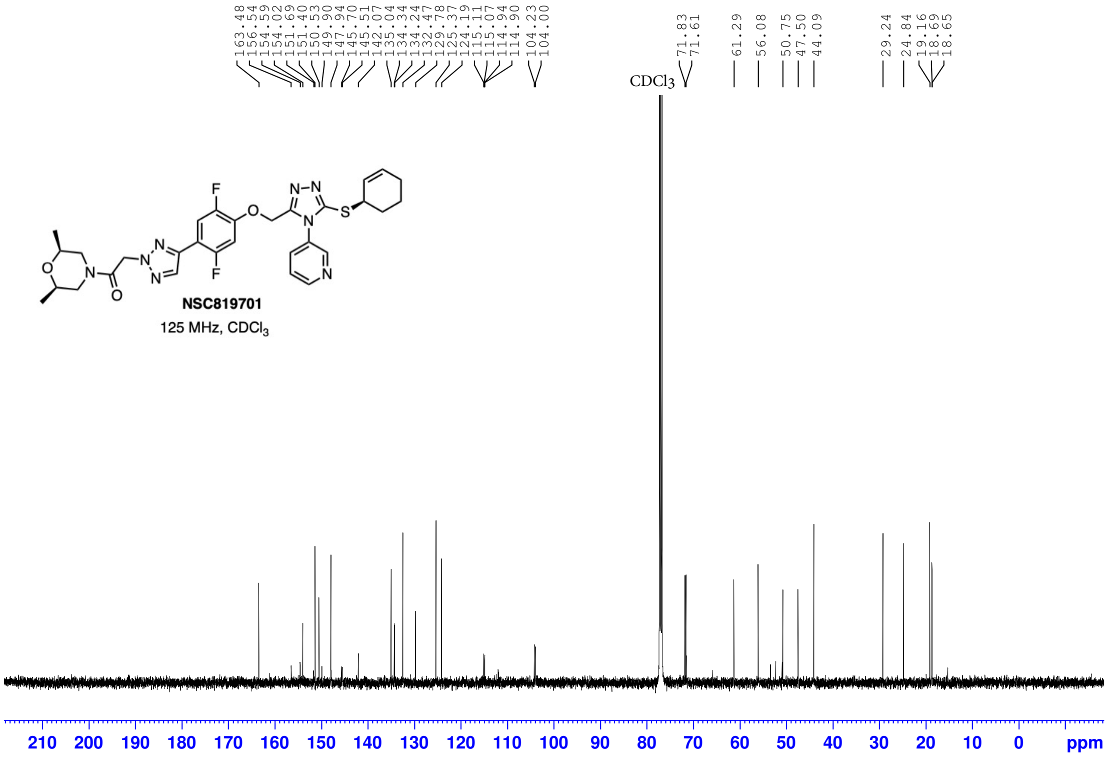
163.48
156.54
154.59
154.02
151.69
151.40
150.53
149.90
147.94
145.70
145.51
142.07
135.04
134.34
134.24
132.47
129.78
125.37
124.19
115.11
115.07
114.94
114.90
104.23
104.00
CDCl3
71.83
71.61
61.29
56.08
50.75
47.50
44.09
29.24
24.84
19.16
18.69
18.65
NSC819701
125 MHz, CDCl3
210 200 190 180 170 160 150 140 130 120 110 100 90 80 70 60 50 40 30 20 10 0 ppm

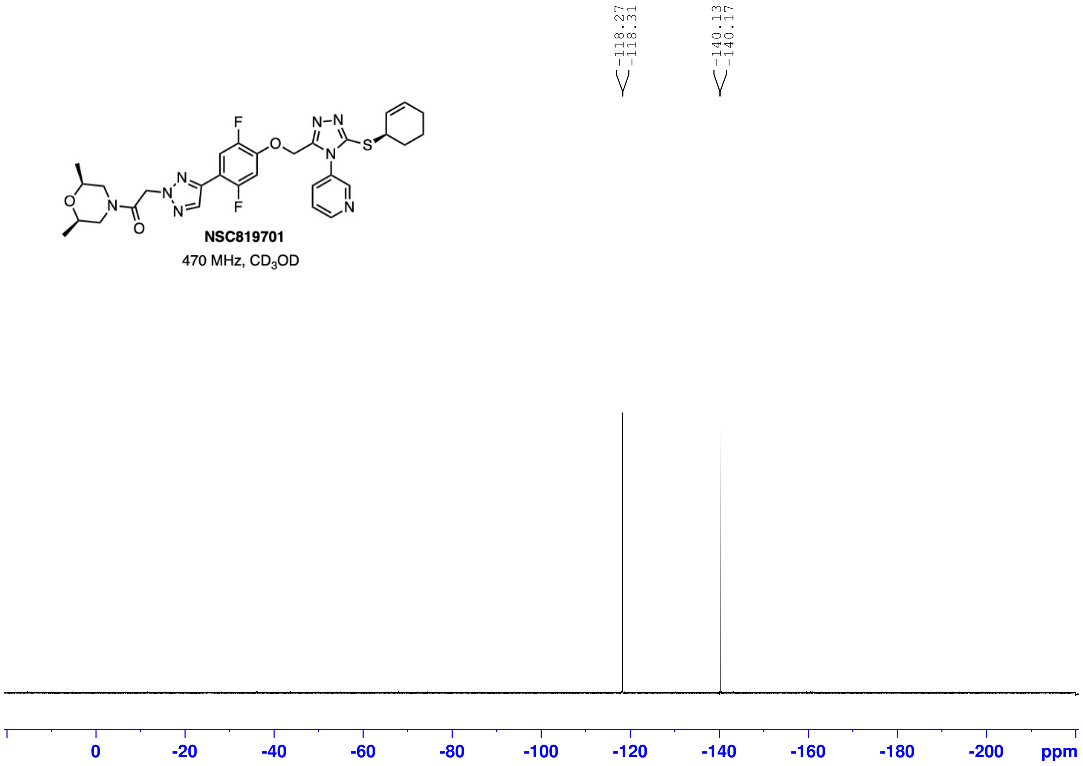

Supplementary Data 3 | Characterization of the triazole compound NSC819701.
